# Designing the future of prenatal care: an algorithm for a telemedicine-enhanced team-based care model

**DOI:** 10.25122/jml-2024-0145

**Published:** 2024-01

**Authors:** Simona Raluca Iacoban, Volodymyr Artyomenko, Madalina Piron-Dumitrascu, Ioan Dumitru Suciu, Luciana Alexandra Pavelescu, Nicolae Suciu

**Affiliations:** 1 Department of Obstetrics and Gynecology, Polizu Clinical Hospital, Carol Davila University of Medicine and Pharmacy, Bucharest, Romania; 2 Department of Obstetrics and Gynecology, Odesa National Medical University, Odesa, Ukraine; 3 Department of Cellular and Molecular Biology and Histology, Carol Davila University of Medicine and Pharmacy, Bucharest, Romania

**Keywords:** prenatal care, innovation, telemedicine, model, algorithm

## Abstract

This study provides a conceptual exploration of an innovative telemedicine-enhanced team-based care (TETC) model, tailored to prenatal care, integrating a multidisciplinary team approach with advanced telemedicine technologies. The algorithm developed for TETC aims to optimize communication and coordination among healthcare professionals, including obstetricians, midwives, nutritionists, and mental health experts. This cohesive team structure ensures a comprehensive care plan encompassing all facets of maternal and fetal health. Leveraging telemedicine tools like video conferencing and digital health records, the model supports remote consultations and coordinated care, proving particularly advantageous during pandemics or in regions with limited healthcare access. Central to the TETC model is patient-centered care, focusing on personalized care plans attuned to the individual needs, health status, and socioeconomic backgrounds of pregnant women. This approach not only enhances accessibility and convenience by diminishing the necessity for physical consultations but also ensures continuity of care throughout pregnancy. This continuity is crucial for consistent health parameter tracking and early risk identification. The paper discusses the model’s design, operational workflow, and ethical and legal considerations, providing implementation guidelines and potential applications. The TETC model, rooted in current technological capabilities and healthcare frameworks, underscores the need for close collaboration with healthcare professionals to adhere to medical standards and address real-world requirements effectively.

## INTRODUCTION

Innovation in prenatal care is crucial for the health of expectant mothers and their newborns. Advancements in this field, such as improved diagnostic tools and digital health platforms, enable early detection of complications and personalized care. These technologies not only enhance the quality of care but also increase its accessibility, substantially improving maternal and neonatal health outcomes worldwide [1–5].

Many studies have emphasized the importance of innovative approaches in maternal and newborn health, particularly in addressing the diverse needs of different populations and contexts [6–9]. For example, a landscape analysis of innovative maternal and newborn health approaches highlighted that most innovations are adaptations of existing interventions to new contexts. These approaches often focus on direct organization and delivery of health services, and include health workforce interventions, health technologies, and novel models of financing and policy making; this study highlighted the need for future implementation and evaluation efforts to assess the effects of innovations on health outcomes and provide evidence on their potential for scale-up, considering cost, feasibility, appropriateness, and acceptability [10].

Another study examined strategies to reduce disparities in the quality of prenatal care in settings with limited resources, highlighting the role of health information technology (IT). Health IT can be used to increase consumer awareness about the importance of preconception and early prenatal care, facilitate spatial mapping of access gaps, improve continuity of patient records, support collaborative quality improvement, and enhance the delivery of preventive health services [11]. Moreover, evidence-based prenatal care is crucial for reducing hospital admissions, improving education, increasing satisfaction, and lowering pregnancy-associated morbidity and mortality. Early initiation of care, identification and treatment of periodontal disease, managing prepregnancy body mass index, and screening for various health conditions are some of the key components of effective prenatal care [12]. The COVID-19 pandemic has also brought important changes to prenatal healthcare delivery, with the integration of telemedicine offering an alternative to exclusively in-office care. This approach has been particularly beneficial for populations in underserved areas or facing access barriers. Despite concerns about access to technology and language barriers in these populations, the uptake of telemedicine did not show meaningful differences according to sociodemographic characteristics, and pregnant women and their healthcare clinicians generally perceived telemedicine positively [13].

We argue that the development and validation of new models and algorithms for delivering integrative prenatal care are essential and could substantially contribute to improved outcomes in maternal and fetal health, especially when tailored to address the specific needs of diverse populations and implemented with a thorough understanding of their potential impact and scalability.

This study is a conceptual exploration in the development of a new model for delivering integrative prenatal care to improve outcomes in maternal and fetal health based on three models: community-based programs, artificial intelligence (AI)-driven care protocols, and telemedicine approaches.

1. Community-based integrative prenatal care model: this could involve a network of healthcare providers, local organizations, and digital platforms to provide comprehensive care. The focus would be on accessibility, education, and continuous support, especially in underprivileged or rural areas. The feasibility of this model lies in its potential for broad impact and adaptability to different community needs.
2. AI-driven personalized prenatal care protocols: using AI to analyze a vast array of data (medical history, current health status, environmental factors) could enable the creation of personalized care plans. This approach could improve outcomes by tailoring interventions to individual needs, but it requires advanced AI algorithms and significant data input.
3. Telemedicine-enhanced team-based care (TETC): this model combines the expertise of various healthcare professionals (obstetricians, midwives, nutritionists, mental health experts) coordinated through a telemedicine platform. It allows for a holistic approach to care, potentially increasing accessibility and continuity of care. This model is quite feasible with current technology and can be scaled according to available resources.

In terms of model development, each of these ideas presents unique challenges: for the community-based model, an algorithm could be developed to optimize resource allocation and identify key areas for intervention based on community health data. This would involve data analysis and predictive modeling. For the AI-driven personalized care, the algorithm would need to be much more complex, involving machine learning to analyze large datasets and generate personalized care plans. Such an algorithm would require a combination of data analysis, machine learning (for risk assessment and predictive modeling), and a robust software infrastructure to support telemedicine consultations and data sharing, therefore it requires extensive data and advanced AI skills. The TETC model would benefit from an algorithm that facilitates efficient communication and coordination among team members, perhaps through a smart scheduling system or an AI-assisted decision support system.

Given the current state of technology and healthcare systems, we chose to develop an algorithm for the TETC model as it seems to be immediately feasible. Of course, collaboration with healthcare professionals is crucial to ensure that the algorithm meets real-world needs and adheres to medical standards and practices.

## MATERIAL AND METHODS

In the development of our innovative prenatal care model, we used a robust conceptual framework that integrates telemedicine with a multidisciplinary approach. The genesis of our algorithm is rooted in a comprehensive review of existing literature, sourced from the latest references available in the Web of Science and PubMed databases, identifying recognized gaps in current prenatal care practices. Our algorithm’s design reflects a systematic amalgamation of theoretical principles and practical considerations, aimed at optimizing patient outcomes through a TETC model. We delineated specific roles and responsibilities for healthcare professionals within the model, ensuring that each discipline’s expertise is effectively used to provide holistic care. In addition, our model incorporates a sophisticated risk assessment mechanism, leveraging algorithmic processing to categorize patients based on their individual health profiles. This stratification guides the subsequent steps in the care pathway, including the scheduling of appointments and tailoring of interventions. The selection of technology and equipment was guided by criteria prioritizing patient safety, data security, and ease of use, ensuring that the model is not only theoretically sound but also pragmatically viable in diverse healthcare settings. Through this meticulous design process, our model aims to revolutionize prenatal care by harmonizing advanced technology with comprehensive, patient-centered care.

## RESULTS

### Core concept

Our TETC model represents a transformative approach to prenatal care, integrating the benefits of telemedicine with the comprehensive support of a multidisciplinary healthcare team. We structured this model around the following core concepts:

1. Multidisciplinary team approach: a cohesive team comprising obstetricians, midwives, nutritionists, and mental health experts collaborates to offer a holistic care package. Each member contributes their specialized knowledge, ensuring a comprehensive care plan that addresses all aspects of maternal and fetal health.
2. Telemedicine integration: using telemedicine technologies, such as video conferencing and digital health records, the model facilitates remote consultations, follow-ups, and coordinated care. This aspect is especially crucial for providing continuous care during situations such as a pandemic or in remote areas where access to healthcare facilities is limited.
3. Patient-centered care: developing personalized care plans that adapt to the unique needs of each pregnant woman, considering their health status, preferences, and socioeconomic background.
4. Accessibility and convenience: by reducing the need for physical visits, the model makes prenatal care more accessible and convenient, especially for those facing geographical, mobility, or time constraints.
5. Continuity of care: ensures consistent and continuous care throughout the pregnancy, improving tracking of health parameters and early identification of potential risks.

### Necessity of the model and addressing gaps in prenatal care

Our model addresses several critical gaps in traditional prenatal care by leveraging the strengths of telemedicine and multidisciplinary collaboration. It offers a forward-thinking solution to enhance accessibility, quality, and continuity of prenatal care, particularly in the face of modern healthcare challenges.

- Improving access to care: traditional prenatal care models often fail to reach marginalized communities or those living in remote areas. Telemedicine bridges this gap, making quality prenatal care more accessible to a broader population.
- Responding to modern challenges: the COVID-19 pandemic has highlighted the need for adaptable healthcare models that can provide uninterrupted care even in crisis situations. Telemedicine is pivotal in such scenarios.
- Comprehensive care coordination: the multidisciplinary nature of the team allows addressing not just the physical aspects of prenatal care but also nutritional, psychological, and social factors, leading to a more rounded approach to maternal and fetal health.
- Reducing healthcare disparities: by making prenatal care more accessible and adaptable, the model can play a significant role in reducing healthcare disparities, especially for underserved populations.
- Enhancing patient satisfaction and engagement: the convenience and continuity of care provided by this model can lead to higher patient satisfaction and engagement, essential components for positive health outcomes.
- Cost-effectiveness: by potentially reducing the need for frequent in-person visits and optimizing resource allocation, this model can be more cost-effective compared to traditional care models.

### Design of the model

Implementing a TETC model requires detailed planning in terms of staff involvement, equipment needs, and knowledge requirements. The framework of the algorithm that would ensure a comprehensive and effective implementation, maximizing the benefits of telemedicine in prenatal care, including how the various healthcare professionals are integrated and the type of technology recommended, is presented in Table 1.

**Table 1:** Framework of the TETC model

Staff requirements	Equipment and technology needs	Knowledge and training requirements	Implementation steps	Monitoring and evaluation
**Medical staff:** obstetricians, gynecologists, midwives, nurses, nutritionists, mental health professionals	**Telemedicine platform:** secure, user-friendly platform for video consultations, data storage	**Medical training:** continued education on prenatal care and telemedicine	**Assessment and planning:** assess capabilities, identify resource gaps, plan for roles and schedules	**Performance metrics:** track patient wait times, consultation effectiveness, satisfaction, health outcomes
**IT and technical** support team: IT specialists, data analysts, training personnel	**Computers and tablets:** high-quality devices for staff to conduct virtual consultations, manage patient data	**Technical training:** training on the use of the telemedicine platform	**Installation and setup:** set up telemedicine platform and technological infrastructure	**Feedback loops:** establish channels for staff and patient feedback
**Support and ancillary staff:** patient coordinators, technical support	**Internet infrastructure:** reliable and high-speed internet connection	**Data privacy and security training:** training on data protection laws and patient confidentiality	**Staff training and onboarding:** conduct training sessions and provide resources	**Continuous improvement:** review system performance, make iterative improvements
	**Data servers:** secure servers for storing patient data and running the algorithm	**Algorithm-specific training:** education on how the algorithm works and its role in patient care	**Pilot testing:** test the system with a small group, gather feedback, adjust	
	**Communication devices:** smartphones or tablets for patients		**Full-scale implementation:** gradually increase usage, continuously monitor performance	
			**Evaluation and adaptation:** regularly evaluate effectiveness, adapt based on feedback	

We propose a multistep operational workflow that aims to bridge the gap between traditional prenatal practices and modern medical advancements. By meticulously structuring each stage of the patient journey, from registration to outcome improvement, our model aims to enhance the efficacy and responsiveness of prenatal care. Table 2 presents the structured, step-by-step process of our model. It outlines each phase of patient interaction, from initial registration to outcome improvement, highlighting the interconnectedness of these stages and the pivotal role of multidisciplinary involvement in enhancing maternal and fetal health outcomes.

**Table 2:** Operational workflow of the TETC model

Step	Description	Connection to the next step
**1. Patient registration**	Patients enter the system and provide initial information	Leads to detailed assessment of the patient’s condition
**2. Initial assessment**	Gathering detailed health information and specific needs	Determines the level of risk and care required
**3. Risk assessment**	The algorithm categorizes patients based on the initial data	Informs the scheduling system for appointment setting
**4. Smart scheduling**	Automated scheduling of appointments based on risk and availability	Results in the first major healthcare provider–patient interaction
**5. Virtual consultation**	Primary interaction between healthcare providers and patients	Determines the need for further care or specialist involvement
**6. Follow-up care**	Scheduling subsequent care and interventions	Could involve other healthcare professionals based on care needs
**7. Multidisciplinary team**	Involvement of various professionals as needed	Provides insights and experiences from care received
**8. Feedback collection**	Collecting input from patients and staff to assess the system	Forms the basis of analytical review of system performance
**9. Data analysis**	Analyzing feedback and performance metrics	Identifies areas for improvement in care and system functionality
**10. Outcome improvement**	Using insights to refine the care process and feedback into smart scheduling	Enhances the initial stages of the process for future patients

Implementing the TETC model within a healthcare setting involves several critical steps, beginning with the setup of the necessary infrastructure. This setup requires careful selection of both hardware and software. Essential hardware includes computers or tablets equipped with high-definition cameras and microphones, ensuring clear and effective communication during teleconsultations. In addition, a reliable and secure internet connection is paramount to support uninterrupted service. In terms of software, the selection of a telemedicine platform should prioritize features such as video conferencing capabilities, integration with electronic health records (EHR), and robust data security measures, ensuring compliance with healthcare regulations.

Training healthcare professionals to proficiently use this system is another key aspect of implementation. This training should encompass not only the technicalities of operating the software and managing online consultations but also focus on best practices for remote patient engagement and communication. Moreover, it should include guidelines on maintaining data security and patient privacy during virtual interactions.

### Potential applications

The potential applications of this model extend to various contexts, including both rural and urban settings. In rural areas, where access to specialized care is often limited, this model can significantly enhance the reach and quality of prenatal care. Conversely, in urban clinics, it can be used to streamline and optimize care delivery amidst high patient volumes. The inherent flexibility of this model allows for customization according to available resources and specific patient needs. For instance, in resource-limited settings, the model can be adapted to use more basic telemedicine tools, while in more resource-abundant settings, it could integrate advanced monitoring technologies.

### Ethical and legal considerations

Ensuring patient privacy and data security is a top priority. The telemedicine system must incorporate strong encryption methods and secure data storage solutions to protect sensitive patient information. Additionally, compliance with legal and ethical standards, including informed consent for telemedicine services and adherence to medical data protection laws, is imperative. Healthcare providers must be well-informed about these aspects and stay updated on evolving regulations in telehealth.

### Successful implementation

**Figure 1:**
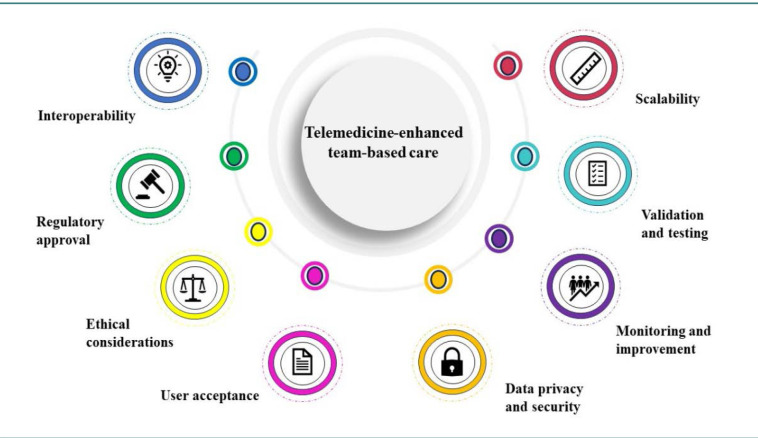
Key considerations for the implementation of the TETC model

There are many aspects that have a critical role in the successful development, implementation, and ongoing management of a healthcare algorithm like the one we proposed for integrative prenatal care (Figure 1):

- Validation and testing: this involves rigorous testing of the model in controlled settings and real-world environments. Clinical trials can assess effectiveness, accuracy, and safety. Real-world evaluations can further validate performance across diverse patient populations and settings.
- Data privacy and security: compliance with data protection laws like the Health Insurance Portability and Accountability Act of 1996 (HIPAA) in the United States or the General Data Protection Regulation (GDPR) in Europe is critical. This includes implementing robust encryption, secure data storage, and controlled access protocols to protect patient information from unauthorized use or breaches.
- Interoperability: the algorithm should be compatible with existing healthcare IT systems and EHRs. This ensures smooth data exchange and integration into current clinical workflows, enhancing efficiency and usability.
- User acceptance: training for healthcare providers is essential to ensure they understand and trust the algorithm. Patient education is equally important to address any concerns and gain their acceptance, which is crucial for the successful adoption of the technology.
- Scalability: the algorithm must be designed to handle an increasing number of users and a growing volume of data without degradation in performance. This ensures that it can be effectively used in various healthcare settings, from small clinics to large hospitals.
- Ethical considerations: it is vital to ensure that the model does not perpetuate existing biases or create new ones. Fairness and transparency in decision-making should be key goals to avoid disparities in healthcare delivery.
- Regulatory approval: obtaining approval from regulatory bodies like the Food and Drug Administration (FDA) in the United States involves demonstrating the algorithm’s safety, effectiveness, and compliance with medical device regulations. This process can be complex and time-consuming but is essential for clinical use.
- Continuous monitoring and improvement: after deployment, the algorithm should be continuously monitored for performance and outcomes. Regular updates and improvements should be made based on new medical research, user feedback, and changing healthcare practices.

## DISCUSSION

The proposed TETC algorithm holds significant potential for advancing the field of prenatal care, particularly from an integrative perspective. This algorithm harmonizes the principles of modern technology with the nuanced needs of prenatal healthcare, offering a comprehensive approach that could revolutionize how prenatal care is delivered.

The integration of telemedicine provides a platform for expanding access to prenatal care, an essential factor in improving maternal and fetal health outcomes. Studies have shown that telemedicine can effectively deliver prenatal care, particularly in rural or underserved areas, addressing issues of accessibility and equity in healthcare [14,15].

A study by Tian *et al*. compared the effectiveness of telemedicine with standard prenatal care for blood glucose control in women with gestational diabetes mellitus. This randomized controlled trial assessed whether health education and lifestyle management delivered through instant messaging platforms such as WeChat was more effective than standard clinical prenatal care in controlling blood glucose among women with gestational diabetes mellitus and found that telemedicine interventions, such as instant messaging platforms, were more effective for blood glucose control compared to standard clinical prenatal care alone [16].

More studies have showed that telemedicine is an effective and feasible method for providing prenatal care in rural and underserved areas, helping to increase equality and accessibility of healthcare services [17–19].

Moreover, the algorithm’s emphasis on a multidisciplinary team approach aligns with the growing recognition of the importance of holistic care in obstetrics. Integrative prenatal care, which includes not only obstetricians but also midwives, nutritionists, and mental health professionals, has been linked to improved maternal and child health outcomes [20–22]. By facilitating seamless collaboration among various healthcare professionals, the algorithm ensures that each patient receives personalized, comprehensive care, addressing a wide range of health determinants.

Furthermore, the algorithm’s capacity for risk assessment and smart scheduling supports proactive and preventive care strategies. Predictive analytics, a key component of the algorithm, can identify high-risk pregnancies early, allowing for timely interventions that can prevent complications [23,24]. This approach not only improves health outcomes but also optimizes the allocation of healthcare resources.

The ability to conduct virtual consultations and monitoring can significantly reduce barriers to care, such as transportation challenges and time constraints, which are critical factors in ensuring consistent and comprehensive prenatal care. One systematic review of 23 articles highlighted that virtual prenatal care offers significant advantages over traditional in-person care when designed with the inclusion of the needs of pregnant women and healthcare professionals. This review found that virtual care can substantially reduce travel time, absences from work, clinic wait times, and no-show rates, while also limiting exposure risks during pandemics and increasing self-accountability [25]. Another study focusing on the acceptability of virtual prenatal and postpartum care among maternity care providers underlines the evolving perceptions and adaptations in healthcare delivery, especially in the context of recent global health crises [26].

Further evidence from a controlled trial involving a mobile prenatal care app demonstrated how such digital interventions could reduce the need for in-person visits, supporting the notion of improved accessibility and efficiency in prenatal care delivery [27]. Moreover, a study on telemedicine use and perceived quality of virtual care among pregnant and postpartum women during the COVID-19 pandemic also provided insights into the shifting landscape of prenatal care, emphasizing the growing role of telehealth and the virtual care model [27].

Feedback collection and data analysis, integral to the algorithm, enable continuous quality improvement. By systematically collecting patient and provider feedback and analyzing care outcomes, the algorithm fosters an adaptive learning system. This approach aligns with evidence-based practices, ensuring that prenatal care evolves in response to emerging research and patient needs [28].

### Limitations of telemedicine

The TETC model, while innovative, faces several limitations that are crucial to consider. One of the primary concerns with virtual prenatal visits is their appropriateness across different stages of pregnancy and for varying risk levels.

A recent study found that virtual visits might be less suitable during the first and third trimesters and for high-risk pregnancies. This is partly due to the need for establishing a patient–doctor relationship and the increased risks as the due date approaches. The authors also stated that the technological aspect can be a significant limitation. Patients have reported issues like the need for better audiovisual connections, resolution of virtual platform technical difficulties, increased bandwidth during high usage periods, and improved real-time customer support [29].

Moreover, a study found that about one in three respondents used prenatal telehealth, with the most common reason for not using it being a personal preference for in-person care. This preference indicates that while telehealth has benefits, it may not be the preferred mode of care for all patients [30].

Lastly, a study from Brazil focusing on low-risk antenatal care enhanced by telemedicine found that while telemedicine can reduce hospital exposure and care costs, there are no validated protocols for prenatal care via telemedicine, indicating a need for more structured guidelines and protocols to ensure the safe and effective use of telemedicine in prenatal care [31].

Other studies have explored the constraints and drawbacks associated with telemedicine and provide a comprehensive examination of the multifaceted challenges of telemedicine [32–35].

### Strengths and limitations

A primary strength of this study lies in its forward-thinking integration of telemedicine into prenatal care, showcasing a model that leverages digital technology to enhance communication and coordination among a multidisciplinary healthcare team. This approach is particularly pertinent in the evolving landscape of digital health, offering a potentially transformative solution for challenges like access barriers and continuity of care during crises such as pandemics. The model’s emphasis on a multidisciplinary team encompassing obstetricians, midwives, nutritionists, and mental health professionals presents a holistic approach to prenatal care, ensuring comprehensive management that addresses diverse aspects of maternal and fetal health. However, the study’s primary limitation is its theoretical nature, lacking empirical validation through longitudinal studies. While the algorithm and the model are well-conceived, the absence of practical implementation data means that the effectiveness, feasibility, and real-world impact of the model remain speculative. This gap underscores the need for future research involving empirical testing and evaluation to substantiate the proposed benefits of the TETC model. Additionally, the reliance on technology and digital platforms could pose accessibility challenges in regions with limited technological infrastructure, potentially affecting the model’s applicability in varied socioeconomic contexts.

## CONCLUSION

The TETC model could have profound implications for prenatal care, offering a more accessible, comprehensive, and adaptive approach. Its implementation could lead to significant advancements in maternal and fetal health, particularly in areas with limited access to traditional healthcare resources. As prenatal care continues to evolve, such integrative and technology-driven approaches will likely become increasingly central to delivering high-quality care. We encourage further research evaluating the validity of the algorithm in several healthcare settings, to better understand its advantages and limitations. Moreover, an adapted form of the algorithm could have applicability beyond prenatal care settings.
